# Isoalantolactone relieves depression-like behaviors in mice after chronic social defeat stress via the gut-brain axis

**DOI:** 10.1007/s00213-023-06413-8

**Published:** 2023-07-03

**Authors:** Siming Wang, Qihan Cai, Lu Xu, Yanan Sun, Mengmeng Wang, Yu Wang, Lili Zhang, Keqing Li, Zhiyu Ni

**Affiliations:** 1 School of Basic Medical Science, Hebei University, Baoding, 071000 Hebei Province People’s Republic of China; 2 College of Traditional Chinese Medicine, Hebei University, Baoding, 071000 China; 3 Hebei Provincial Mental Health Center, Baoding, 071000 Hebei Province China; 4 Hebei Key Laboratory of Major Mental and Behavioral Disorders, Baoding, 071000 China; 5 Baoding, China; 6 Affiliated Hospital of Hebei University, Baoding, 071000 China; 7 Clinical Medical College, Hebei University, Baoding, 071000 Hebei Province People’s Republic of China; 8 Hebei Collaborative Innovation Center of Tumor Microecological Metabolism Regulation, Baoding, 071000 China

**Keywords:** Short-chain fatty acids, Isoalantolactone, CSDS, Inflammation, Gut microbiota

## Abstract

**Rationale:**

The management of depression continues to be challenging despite the variety of available antidepressants. Herbal medicines are used in many cultures but lack stringent testing to understand their efficacy and mechanism of action. Isoalantolactone (LAT) from Elecampane (*Inula helenium*) improved the chronic social defeat stress (CSDS)-induced anhedonia-like phenotype in mice comparable to fluoxetine, a selective serotonin reuptake inhibitor (SSRI).

**Objectives:**

Compare the effects of LAT and fluoxetine on depression-like behaviors in mice exposed to CSDS.

**Result:**

The CSDS-induced decrease in protein expression of postsynaptic density (PSD95), brain derived neurotrophic factor (BDNF), and glutamate receptor subunit-1 (GluA1) in the prefrontal cortex was restored by LAT. LAT showed robust anti-inflammatory activity and can lessen the increase in IL-6 and TNF-α caused by CSDS. CSDS altered the gut microbiota at the taxonomic level, resulting in significant changes in α- and β-diversity. LAT treatment reestablished the bacterial abundance and diversity and increased the production of butyric acid in the gut that was inhibited by CSDS. The levels of butyric acid were negatively correlated with the abundance of *Bacteroidetes*, and positively correlated with those of *Proteobacteria* and *Firmicutes* across all treatment groups.

**Conclusions:**

The current data suggest that, similar to fluoxetine, LAT show antidepressant-like effects in mice exposed to CSDS through the modulation of the gut-brain axis.

**Supplementary information:**

The online version contains supplementary material available at 10.1007/s00213-023-06413-8.

## Introduction

The prevention and treatment of depression are worldwide concerns. As the pace of modern life continues to accelerate, people are under increasing pressure in their professional and personal lives, resulting in rising incidences of depression over the years (Yrondi et al. 2017). Recently, the global new coronavirus (COVID-19) outbreak in 2020 saw the prevalence of depression reaching a record high, particular in adolescents, who showed the worst depression and anxiety symptoms (Varma et al. 2021). Despite great advances in the understanding of depression and its underlying mechanisms, current available antidepressants still present significant limitations including low efficacy, drug resistance, and severe adverse reactions (Vázquez et al. 2021). Therefore, the development of new, and preferably natural, drugs for the treatment of depression is of great importance and significance.

Depression is a complex disease with multiple mechanisms involving numerous neurotransmitters and biochemical substances, various brain regions and circuits, and the immune system (Price and Duman 2020). The gut-brain axis has been shown to play an important role in the pathogenesis of stress-related diseases, especially in the phenotypes of depression and anhedonia in rodents (Chang et al. 2022). Abnormal composition of the intestinal microbiome may also contribute to depression and other diseases (Kesika et al. 2021). This may be due to the close relationship between intestinal flora and peripheral immunity. Inflammatory factors can enter the nervous system via different pathways to activate microglia cells and affect the expression of depression-related proteins, thus aggravating or improving depression symptoms (Zhou et al. 2020). Patients with depression were found to have disrupted intestinal bacteria composition, reduced abundance and diversity, and relatively less butyrate metabolite-producing flora (Zheng et al. 2016). Fecal microbes from depressed patients can induce depression-like behaviors in rats in fecal transplant experiments (Friedrich 2015). These studies suggest a relationship between the gut microbiota and the regulation of depression symptoms.

There is substantial evidence linking depression with increased immune system activity (Howren et al. 2009). Inflammatory cytokines such as C-reactive protein (CRP), tumor necrosis factor-α (TNF-α), and interleukin-6 (IL-6) are increased in depressed patients compared with healthy controls and may be associated with differential response to antidepressant treatment (Köhler et al. 2017; Osimo et al. 2020; Uher et al. 2014). Based on these etiological findings, some researchers have proposed that anti-inflammatory therapy may produce antidepressant effects (Tyring et al. 2006). Clinical trials have found that anti-inflammatory therapy has positive effects on depression (Akhondzadeh et al. 2009; Tyring et al. 2006), and recent meta-analysis supports the same notion, providing additional avenues of research and development of new antidepressant drugs (Kohler et al. 2016).

Studies have shown that Chinese herbal medicines can have anxiolytic and anti-depressant impact (Jiang et al. 2018; Song et al. 2021; Xu et al. 2016), although their mechanisms of action remain unclear. Elecampane (*Inula helenium*) is a perennial herb of the genus *Inula* in the family Asteraceae. Pharmacological studies have demonstrated that sesquiterpene lactones, a secondary metabolite often found in Asteraceae, can suppress the proliferation of tumor cells and have anti-mycobacterial effects (Dang et al. 2020; Xz et al. 2021; Yan et al. 2020). Elecampane has also been shown to contain sesquiterpene lactones in their roots, with similar anti-tumor, anti-inflammatory and anti-fungal activities (Gierlikowska et al. 2020). Sesquiterpene lactones are divided into several groups, of which isoalantolactone (LAT) has been shown to have no cytotoxicity in previous experiments (Ya-Ru et al. 2010).

Given the reported anti-inflammatory properties of sesquiterpene lactones, we postulate that they may also have anti-depressive activity. Here we show that LAT improved the anhedonia-like phenotype in a mouse model of CSDS. To further investigate the potential neuro-immune-endocrine mechanisms involved in the action of LAT, serum inflammatory cytokines and expression of depression-related proteins in the PFC were measured. Finally, the intestinal flora was evaluated, and correlation analysis was performed to clarify the involvement of the microbiome-gut-brain axis in the antidepressant-like effects of LAT.

## Materials and methods

### Animals

Adult male C57BL/6 mice (*n* = 28; age 8 weeks; bodyweight 21–33 g) and CD1 mice (*n* = 20; age 10 weeks; bodyweight > 35 g) were purchased from Beijing Wister River Experimental Animal Science and Technology Co., Ltd., China. Mice were housed at 22 ± 1 °C, 60% humidity, 12-h light/dark cycle (lights on at 8: 00 a.m.), with unrestricted access to drink and food. Euthanasia was performed by cervical dislocation under deep anesthesia with isoflurane. All experiments were conducted in compliance with the National Institutes of Health’s Guidelines for the Care and Use of Experimental Animals as authorized by the Hebei Medical University’s Local Animal Use Committee.

### Drugs and drug administration

Fluoxetine hydrochloride (Flu; Cayman 14,418, USA) was dissolved in distilled sterile saline and used at a final concentration of 10 mg/kg as described previously (Li et al. 2021; Shu et al. 2019). LAT, supplied by the Hebei Medical University School of Pharmacy, was dissolved in 0.5% (w/v) sodium carboxymethyl cellulose (CMC) and administered at a final concentration of 10 mg/kg. The negative group (neg) received 0.5% CMC only (10 mL/kg). All treatments were administered by oral gavage (o.g.).

### Experimental design

**Fig. 1:**
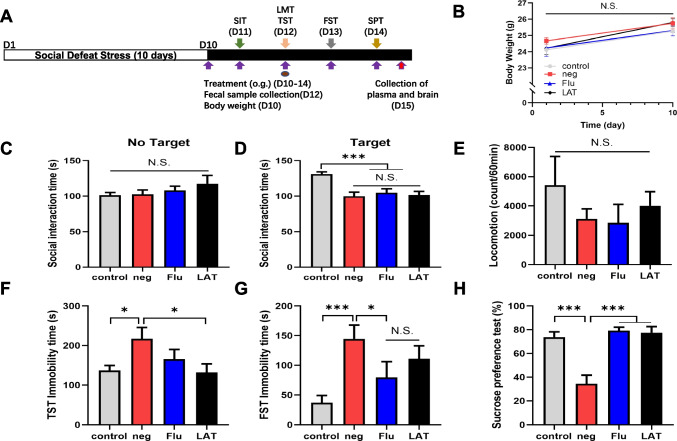
Effects of LAT treatment on depressive behavior. **A** Schema of treatment protocol. Mice were exposed to CSDS for 10 days, followed by drug treatment, behavioral tests (social interaction test (SIT); locomotion (LMT); forced swimming test (FST); tail suspension test (TST); sucrose preference test (SPT)), and sample collection. **B** Graph comparing the body weight of mice on days 0 and 10 (time: *F*_3,24_ = 24.724, *P* < 0.001, group: *F*_3,24_ = 1.214, *P* = 0.326, interaction: *F*_3,24_ = 0.255, *P* = 0.857). Graphs showing the social interaction time **C** without target (*F*_3,24_ = 1.006, *P* = 0.407) and **D** with target (*F*_3,24_ = 9.873, *P* < 0.001). **E** Graph showing the number of movements during the LMT (*F*_3,24_ = 0.733, *P* = 0.542). Graph showing the immobility time during **F** TST (*F*_3,24_ = 3.275, *P* = 0.038) and **G** FST (*F*_3,24_ = 5.186, *P* = 0.007). **H** The percentage of sucrose solution consumed relative to total fluid intake (*F*_3,24_ = 15.853, *P* < 0.001). One-way ANOVA was used for all analyses with interaction scores and *P* values presented (Mean ± S.E.M, *P < 0.05, **P < 0.01, ***P < 0.001. N.S.: not significant)

In a model of CSDS, C57BL/6 mice were exposed to different CD1 aggressor animals for 10 min a day, for 10 days (days 1–10) as previously described (Wang et al. 2021, 2020a, b; Yang et al. 2017). In the 24 h after CSDS, resident CD1 mice and invader C57BL/6 mice were kept on opposing sides of the cage, divided by a perforated plexiglass divider that allowed for visual, olfactory, and aural interaction. After the final stressor on day 10, all mice were housed separately for 24 h. On days 10–14, mice were administered with Flu, LAT, or CMC by oral gavage once a day at 8 am for 5 days. The four experimental groups: (i) control (*n* = 8): no CSDS; (ii) Neg (*n* = 7): CSDS + 5% CMC (10 mL/kg); (iii) Flu (*n* = 6): CSDS + Flu (10 mg/kg); (iv) LAT (*n* = 7): CSDS + LAT (10 mg/kg). Five behavioral tests were performed between days 11 and 14: social interaction (SIT; day 11); locomotion (LMT; day 12); tail suspension (TST; day 12); forced swim (FST; day 13); 1% sucrose preference (SPT; day 14). Fecal samples were taken on day 12. On day 15, the plasma samples were prepared by centrifuging the blood samples at 3000 × g for 3 min. Before analysis, plasma samples are frozen at − 80 °C. Brain samples from the PFC were collected under anesthesia with 30% urethane and stored at − 80 °C until use (Fig. 1A).

### Behavioral tests

#### Social interaction test (SIT)

A social interaction test (SIT) determines whether mice are prone to CSDS. Mice were placed in a 42 × 42 cm^2^ interaction test box with an empty wire-mesh cage (11 × 3.5 cm^2^) at one end. Mice were observed for 2.5 min before and after being exposed to an unknown aggressor housed in a wire-mesh cage for a total of 2.5 min. The amount of time the test subject remains within the “interaction zone,” an 8 cm wide region around the wire-mesh cage housing the aggressor, was recorded. Time spent with an aggressor divided by time spent without one defines the interaction ratio, with a ratio of 1 being the threshold for acceptance. Mice scoring below 1 were considered to be more vulnerable to the effects of social defeat stress, whereas those scoring above 1 reflected abilities to recover more rapidly.

#### Locomotion (LMT)

Mice were housed in a 40 cm squared white plexiglass arena and tracked with the Noldus video tracking software (Wageningen, Netherlands) to evaluate the extent of movement inside the arena for 60 min. The arena was sanitized with 75% alcohol (v/v) between sessions.

#### Tail suspension test (TST)

A four-chambered white plexiglass arena (20 cm × 20 cm × 35 cm), with dividers to prevent interaction between mice, was used for this test. Individual mice were suspended from the suspension bar by their tails, 3 cm from the top of each chamber, for 10 min. Mouse movements were videoed and evaluated using the Noldus video tracking software (Wageningen, Netherlands). Immobility was defined as passive suspension with lack of limb or body movement, and documented for the last 9 min of the recording. The arena was sanitized with 75% alcohol (v/v) between sessions.

#### Forced swim test (FST)

A transparent cylinder (23 cm height × 10 cm diameter) filled with 15 cm of warm water (23 ± 1 °C) was used to monitor the forced swim behavior. Each mouse was placed in the cylinder, and their movements were recorded for 6 min. Immobility, lack of body or limb movement except for those required to stay afloat, was scored using the Noldus video tracking software for the first five minutes.

#### Sucrose preference test (SPT)

Mice were given a 1% sucrose solution for 48 h, fasted for 4 h, and offered a choice of water or 1% sucrose from two identical bottles for one hour. The water and sucrose bottles were weighed before placing in the cage. The proportion of sucrose solution consumed relative to total liquid intake was used to determine whether there is a preference for sucrose.

### Inflammatory cytokine IL-6 and TNF-α measurement

Precoated ELIZA kits were used to detect plasma IL-6 (RK00008; ABclonal, USA) and TNF-α (RK00027; ABclonal, USA) according to the manufacturer’s instructions.

### Western blot analysis

PFC tissues were lysed and extracted using the minute total protein extraction kit (SD-001; Invent Biotechnologies Inc., USA) and quantified by a BCA protein assay kit (PC0020; Solarbio). Aliquots (60 µg) of protein were incubated for 5 min at 95 °C with a quarter volume of denaturing buffer (125 mM Tris–HCl; 20% glycerol; 10% β-mercaptoethanol (w/v); 0.1% bromophenol blue; 4% sodium dodecyl sulfate (w/v); pH 6.8) and subjected to sodium dodecyl sulfate–polyacrylamide gel electrophoresis (SDS-PAGE) using mini-gels (F11420L; ACE). The blots were incubated with primary antibodies against BDNF (SJ12-09 1: 500 dilution; HUABIO, China), GluA1 (SD2010 1: 500 dilution; HUABIO, China), PSD-95 (SR38-09; 1: 500 dilution; HUABIO, China), and β-actin (1:10,000 dilution; AC026; ABclonal, USA) overnight at 4 °C. Visualization was performed with goat anti-rabbit DyLight 800 secondary antibody (1:1000 dilution; S9002, Report) at room temperature for 1 h and imaged with a fluorescence imaging system. Image analysis was performed with Image J 1.0 (https: //imagej.nih.gov/ij/).

### 16S rRNA analysis

Bacterial 16S rRNA genes V3-V4 area using forward primer 338F for polymerase chain reaction (PCR) amplification (5′-ACTCCTACGGGAGGCAGCA-3′) and the reverse primer 806R (5′-GGACTACHVGGGTWTCTAAT-3′). Multiplex sequencing with 7-bp barcoded primers was performed by Shanghai Personal Biotechnology Co., Ltd. on the IllluminaNovaSeq platform. The NovaSeq 6000 SP Reagent Kit (500 cycles) was used for pair-end 2 × 250 bp sequencing of the pooled amplicons after individual quantification (Shanghai, China). Bioinformatic analysis of the microbiome data was conducted with QIIME2 2019.4 (Bolyen et al. 2019), with a minor adjustment made in accordance with the official tutorials (https://docs.qiime2.org/2019.4/tutorials/).

### Measurement of short-chain fatty acid (SCFA) levels in fecal samples

Fresh fecal samples were collected from each mouse at around 10:00 h, upon transfer to a new cage, to avoid circadian effects on the microbiome. Samples were collected and stored in a sterile microtube at − 80 °C until use. SCFA levels were determined by Shanghai Personal Biotechnology, Co., Ltd. (Shanghai, China) according to previous published techniques (Wang et al. 2020b; Zhang et al. 2017). GC–MS/MS analysis of the SCFAs was performed on an Agilent 7890B gas chromatograph with DB-FFAP columns (30 m length × 0.25 mm × 0.25 μm film thickness, J&W Scientific, USA). SCFA data is reported as a milligram per gram of stool (Zhao et al. 2006, 2017).

### Statistical analysis

Data are expressed as the mean ± standard error of the mean (SEM). Analysis software used includes QIIME2 (2019.4) and R script. The statistical analysis was performed using SPSS Statistics 20 (SPSS, Tokyo, Japan). A test of homogeneity of variance for all animal data showed no significant difference. Data of body weight were analyzed using repeated two-way analysis of variance (ANOVA), followed by post hoc Fisher’s least significant difference’s multiple comparison test. Comparisons among four groups were performed using one-way ANOVA followed by Fisher’s LSD test or the Kruskal–Wallis test, followed by the Mann–Whitney U-test. Data for alpha diversity of the gut microbiota were analyzed using the Kruskal–Wallis test, followed by the Mann–Whitney U-test. The algorithm for linear discriminant analysis effect size (LEfSe) was used to analyze microbiota abundance and distribution (Segata et al. 2011). β-diversity was analyzed by principal coordinates analysis (PCoA). The correlation between the number of microorganisms in the gut and the production of SCFAs was analyzed using Spearman’s method. *P* values below 0.05 were considered to be statistically significant.

## Results

### Effects of LAT on depression-like phenotypes

The CSDS protocol was well tolerated by the mice as no significant changes in body weight were observed (Fig. 1B). All mice exposed to CSDS showed depression-like symptoms and impaired social interaction (Fig. 1 C, D), but motor skills were unaffected (Fig. 1E). As shown in Fig. 1F, the results of one-way ANOVA revealed significant differences between groups in the immobility time (*F*_3,24_ = 3.275, *P* = 0.038). Post hoc comparisons indicated that compared with the neg group, LAT administration effectively reversed the CSDS-induced prolonged immobility time (LAT: *P* < 0.05). In FST (*F*_3,24_ = 5.186, *P* = 0.007), only a decrease in immobility time was observed with Flu compared with the neg group (Flu: *P* < 0.05), no difference was observed between Flu and LAT (1G). In addition, SPT (*F*_3,24_ = 15.853, *P* < 0.001). Sucrose preference was significantly reduced in the neg group compared to the control group, and treatment with Flu and LAT normalized sucrose preference compared to the negative control group (Flu: *P* < 0.001, LAT: *P* < 0.001). These results provide the first indication that LAT has antidepressant effects that may be comparable to Flu.

### Effects of LAT on plasma inflammatory cytokines and prefrontal cortex proteins

**Fig. 2:**
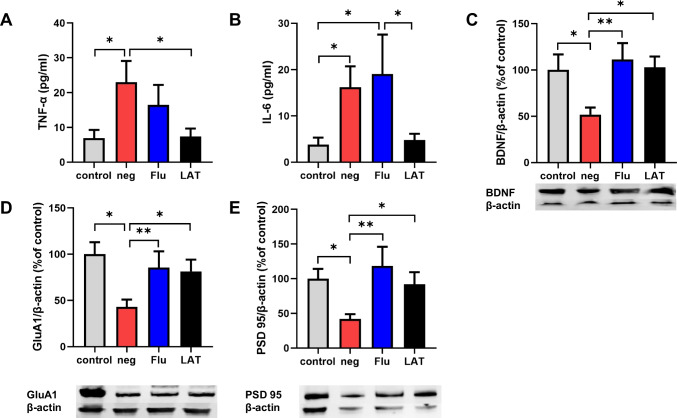
Effects of LAT treatment on serum cytokine and prefrontal cortex protein levels. Graphs showing the plasma level of **A** TNF-α (*F*_3,24_ = 3.358, *P* = 0.035) and **B** IL-6 (*F*_3,24_ = 3.120, *P* = 0.045) concentration in each treatment group. Representative Western blots and summarized graphs showing expression of **C** BDNF (*F*_3,24_ = 3.661, *P* = 0.027); **D** GluA1 (*F*_3,24_ = 3.646, *P* = 0.027); **E** PSD-95 (*F*_3,24_ = 3.521, *P* = 0.030). One-way ANOVA was used for all analyses with interaction scores and *P* values presented (Mean ± S.E.M, *P < 0.05, **P < 0.01, ***P < 0.001)

We evaluated plasma IL-6 and TNF-α levels as these cytokines were previously shown to be increased in CSDS mice (Szyszkowicz et al. 2017; Wang et al. 2021, 2020a, 2020b). Indeed, this observation was reproducible in the neg group of our experiment. As shown in Fig. 2A, B, the results of one-way ANOVA revealed significant differences between groups on the levels of IL-6 and TNF-α (*F*_3,24_ = 3.358, *P* = 0.035, *F*_3,24_ = 3.120, *P* = 0.045). Post hoc comparisons indicated that LAT treatment significantly lowered the levels of IL-6 and TNF-α compared to the neg group and Flu group (LAT: *P* < 0.05), essentially restoring these cytokines to control levels (Fig. 2A, B). Flu treatment, on the other hand, did not affect the expression of these cytokines significantly. For the PFC proteins GluA1, BDNF, and PSD-95, the results of one-way ANOVA revealed significant differences between groups’ effects on protein levels in GluA1 (*F*_3,24_ = 3.646, *P* = 0.027), BDNF (*F*_3,24_ = 3.661, *P* = 0.027), and PSD-95 (*F*_3,24_ = 3.521, *P* = 0.030), and CSDS significantly downregulated their expression, while post hoc comparisons indicated that Flu and LAT administration effectively reversed CSDS-induced PFC damage (Flu: *P* < 0.01, LAT: *P* < 0.05, Fig. 2 C–E). No significant difference was observed between Flu and LAT. These data suggest that LAT has a similar or better effect than fluoxetine in the treatment of anhedonia-like phenotype, inflammation, and PFC depression-related proteins that are altered in CSDS-induced mice.

### Effects of LAT on gut microbiota

**Fig. 3:**
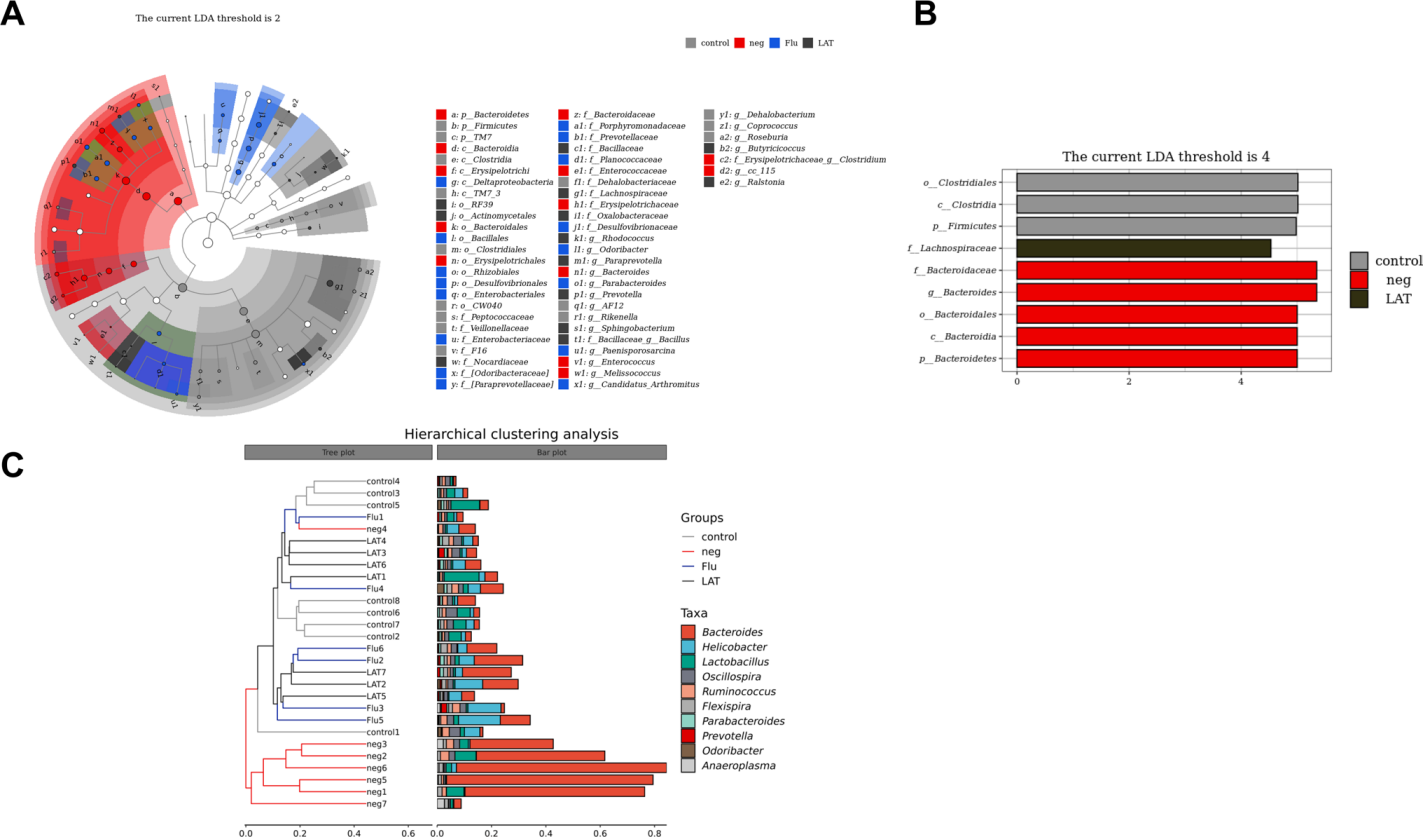
Effects of LAT treatment on the gut microbiota analyzed by the LEfSe algorithm and hierarchical clustering. **A** Taxonomic cladogram generated by LEfSe analysis illustrating significant shifts in the gut microbiota of mice in all groups. From the inner ring to the outer ring, each successive circle represents a taxonomic branch with rich differences at the phylum, class, order, family, genus, and species levels. **B** Histograms of the most abundant taxa based on linear discriminant analysis score cutoff values (log10) > 4.0 and *P* < 0.05 showing significant differences in abundance among the control, neg, and LAT groups. **C** Hierarchical cluster tree diagram showing the similarity between different samples

Previous study showed that CSDS can induce abnormalities in the gut microbiota community (Qu et al. 2017; Zhang et al. 2017). Here, we analyzed the microbiota abundance with LEfSe and found significant variability among the four treatment groups (Fig. 3A). Interestingly, Bacteroides were found to be the dominant genus in the neg group (Fig. 3B). Hierarchical clustering analysis showed that the neg group was distinct from the control, while the Flu and LAT groups clustered closer to control (Fig. 3C).

**Fig. 4:**
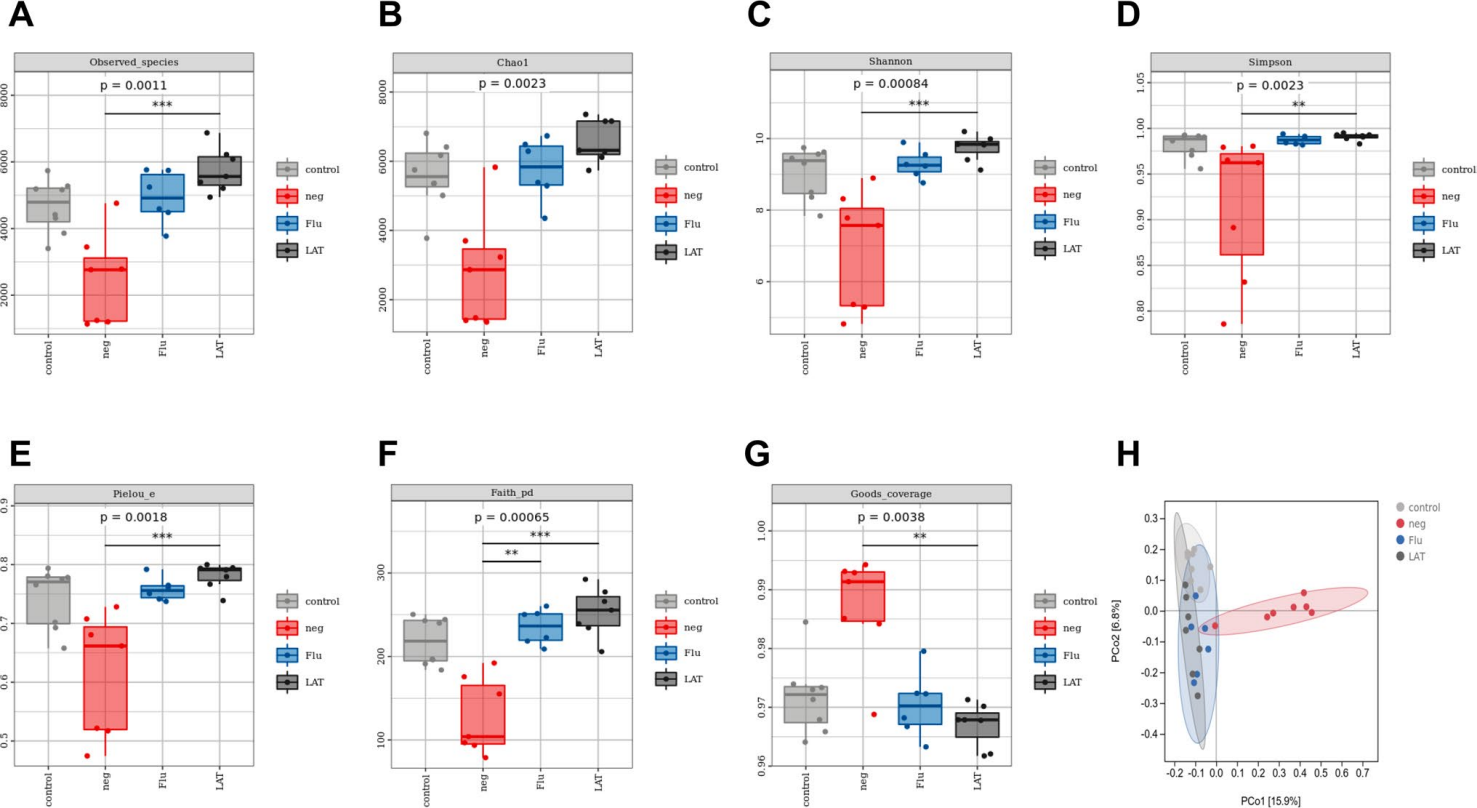
Effects of LAT treatment on the α- and β-diversity of the gut microbiota. Graphs showing the α-diversity analyzed with different indexes: **A** observed (*χ*^2^ = 16.073, *P* = 0.001); **B** Chao1 (*χ*^2^ = 14.482, *P* = 0.002); **C** Shannon (*χ*^2^ = 16.638, *P* = 0.001); **D** Simpson (*χ*^2^ = 14.523, *P* = 0.002); **E** Pielou (*χ*^2^ = 15.056, *P* = 0.002); **F** Faith (*χ*^2^ = 17.180, *P* = 0.001); **G** Goods (*χ*^2^ = 13.434, *P* = 0.004). The Kruskal–Wallis test was used for all analyses with interaction scores and *P* values presented (Mean ± S.E.M,*P < 0.05, **P < 0.01, ***P < 0.001). **H** Based on the unweighted UniFrac PCoA graph of the distance (ANOSIM, *R* = 0.5085, *P* = 0.001). Each point represents a single sample with 15.9% of the *X*-axis principal component and 6.8% of the *Y*-axis principal component

Analysis of the α-diversity for the four treatment groups showed that CSDS treatment significantly reduced the diversity compared to control, while Flu and LAT treatments restored diversity to control levels. This was observed in all indexes: observed, Chao-1, Shannon, Simpson, Pielou_e, Faith_pd, and Goods_coverage (Fig. 4A–G). For β-diversity, PCoA analysis indicated a significant separation of community composition in the neg group (*R* = 0.5085, *P* = 0.001), with the Flu and LAT groups again clustering close to the control (Fig. 4H).

**Fig. 5:**
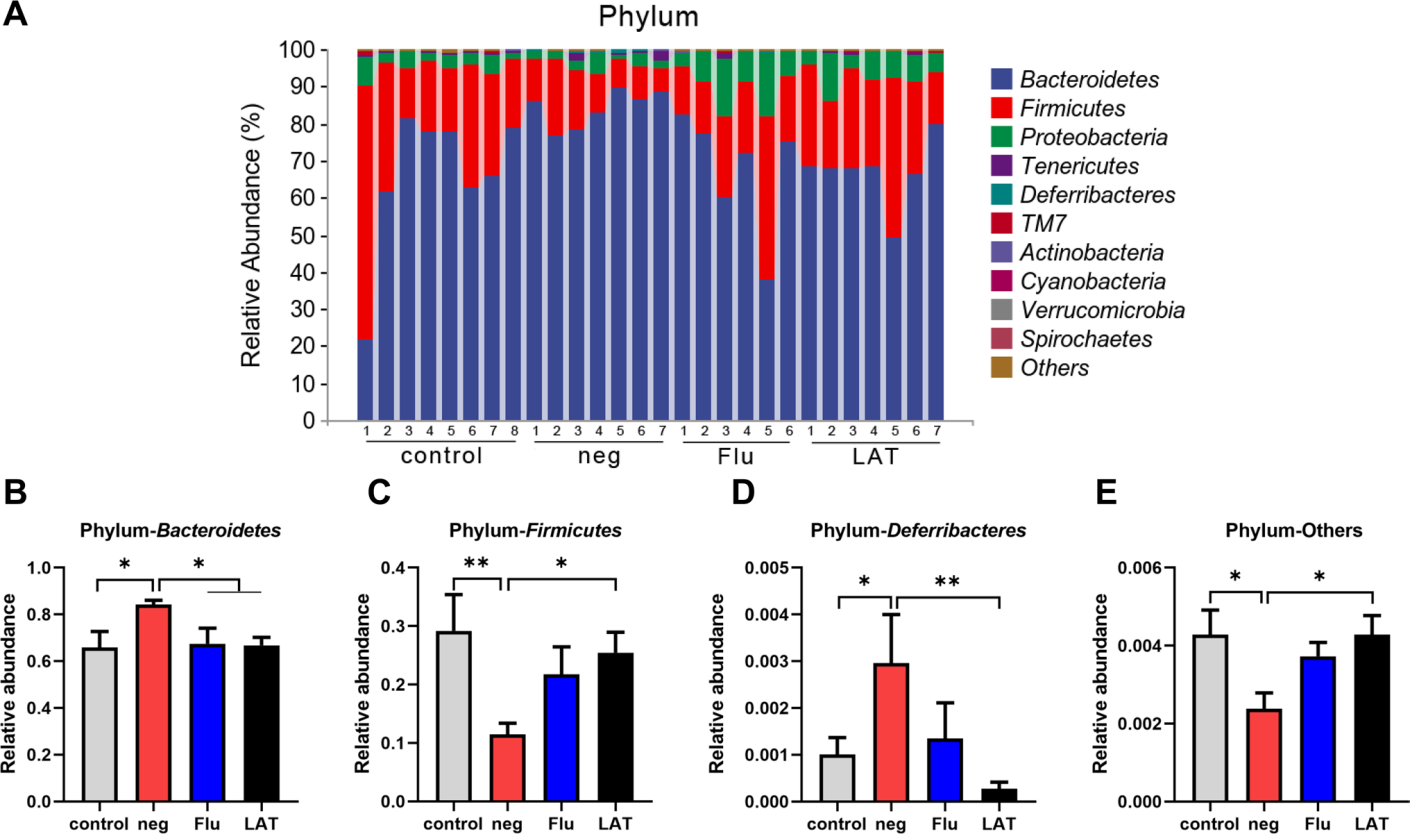
Effects of LAT treatment on the composition of gut microbiota at the phylum level. **A** Graph showing the relative abundance of the phyla identified in fecal samples of all four treatment groups. Graph comparing the relative abundance of **B** *Bacteroidetes* (*χ*^2^ = 10.340, *P* = 0.016); **C** *Firmicutes* (*χ*^2^ = 8.146, *P* = 0.043); **D** *Deferribacteres* (*χ*^2^ = 17.980, *P* = 0.0004); **E** Phyla that were grouped as others (*χ*.^2^ = 3.228, *P* = 0.040). The Kruskal–Wallis test was used for all analyses with interaction scores and *P* values presented (Mean ± S.E.M, *P < 0.05, **P < 0.01, ***P < 0.001)

At the phylum levels, varying abundance can be observed among the four treatment groups (Fig. 5A). In particular, *Bacteroidetes* and *Deferribacteres* were significantly increased in CSDS-treated mice (neg group), while *Firmicutes* and others were reduced. In all cases, Flu and LAT treatments were able to return the respective phylum to the near control level (Fig. 5B–E).

### Short-chain fatty acid (SCFA) concentration and bacterial abundance correlations

**Fig. 6:**
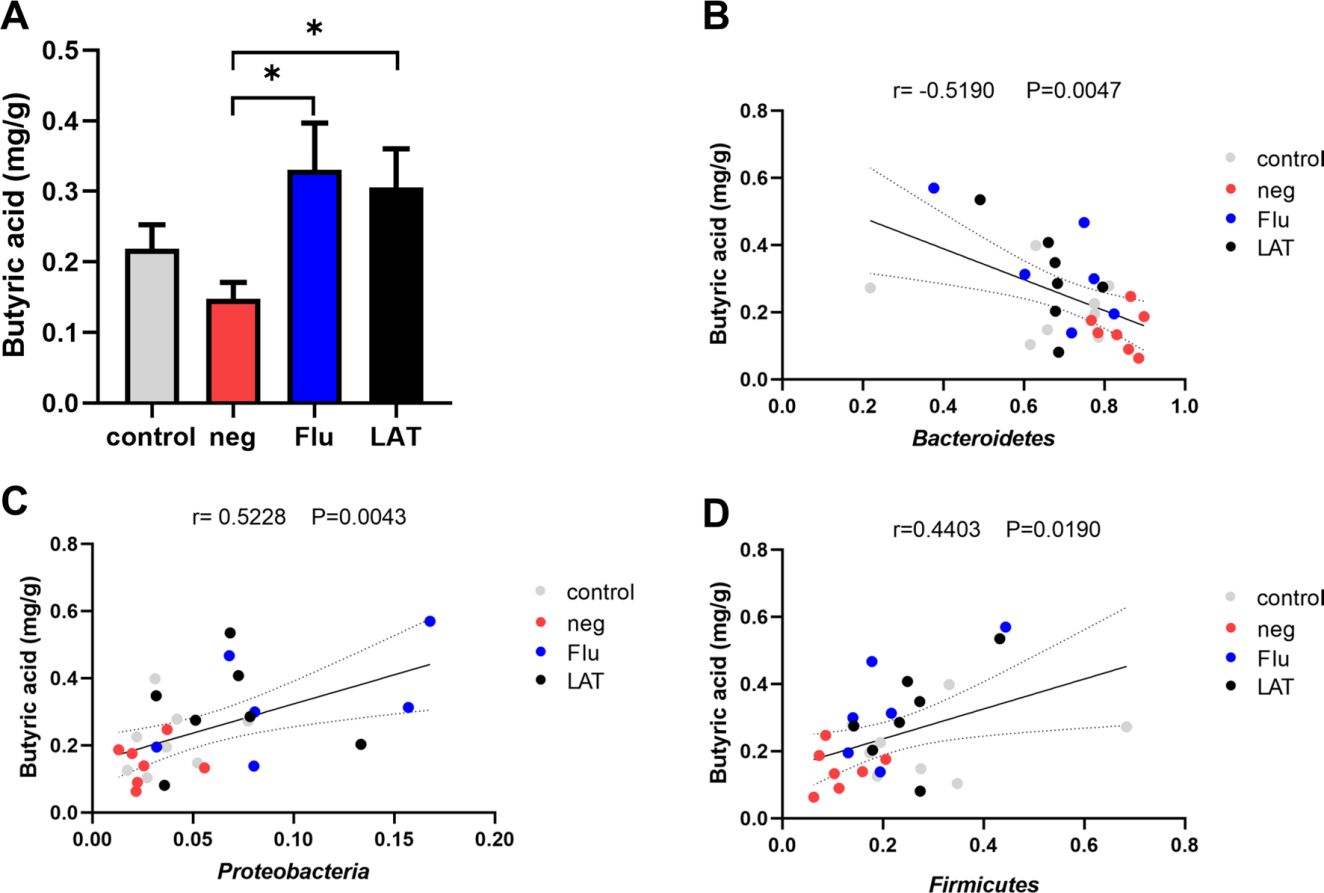
SCFAs levels in fecal samples and their association with bacterial abundance. **A** Graph showing the levels of butyric acid in fecal samples from the four treatment groups (one-way ANOVA, *F*_3,24_ = 3.233, *P* = 0.040). Graphs showing correlation between butyric acid concentration and abundance of **B** *Bacteroidetes* (*R* =  − 0.5190, *P* = 0.0047); **C** *Proteobacteria* (*R* = 0.5228, *P* = 0.0043); **D** *Firmicutes* (*R* = 0.4403, *P* = 0.0190) (Mean ± S.E.M, *P < 0.05, **P < 0.01, ***P < 0.001)

Measurements of SCFA concentrations in fecal samples revealed that CSDS reduced the butyric acid level compared to control, while Flu and LAT treatment significantly increased the acid levels (Fig. 6A). Putative association between the SCFA levels and relative bacterial abundance in fecal samples was investigated using logistic regression and correlation analysis. At the phylum level, *Bacteroidetes* showed a negative correlation with butyric acid (*R* = -0.519, *P* = 0.0047), while *Proteobacteria* (*R* = 0.5228, *P* = 0.0043) and *Firmicutes* (*R* = 0.4403, *P* = 0.019) were positively correlated (Fig. 6B–D).

## Discussion

Depression is a complex disease that still lacks long-term effective treatment. Using a mouse model of CSDS-induced depression we showed that the sesquiterpene lactone, LAT, can reverse depression-like behaviors in a number of behavioral tests, similar to the effect of the clinical anti-depressant fluoxetine. Improvements in behavior were accompanied by the reversal of CSDS-induced changes in serum inflammatory cytokines, prefrontal cortex proteins, and gut microbiota, indicating that the anti-depressive action of LAT is related to the gut-brain axis.

LAT was previously shown to have anticancer activity against various tumors (Huang et al. 2021; Lu et al. 2018; Ya-Ru et al. 2010) and inhibits bacterial endotoxin lipopolysaccharide (LPS)-induced inflammation (Ding et al. 2019; Song et al. 2021). LPS is known to cause depression-like behaviors and dysbiosis of gut microbiota (Ma et al. 2022a, b; Zhang et al. 2020). Peripheral administration of LPS can induce rodent depression-like behaviors following inflammation (Dantzer et al. 2008; O'Connor et al. 2009; Remus and Dantzer 2016), suggesting a possible causal relationship between inflammation and depression. The current observation in our study that LAT reduces CSDS-induced increase in IL-6 and TNF-α, concomitant with the mitigation of depression-like behavior, strongly supports this hypothesis.

The enteric nervous system (ENS) is the largest nerve organ outside the brain and can operate largely autonomously, responding to and adapting to local responses (De Schepper et al. 2018). The ENS includes sensory neurons, motor neurons, and interneurons, which jointly drive secretory function, detect lumen contents, and control intestinal peristalsis (Kuswanto et al. 2016). The gut is also a large lymphatic organ (Gabanyi et al. 2016), and a close relationship between the immune system and the intestinal nervous system has been demonstrated (Huh and Veiga-Fernandes 2020). The intestinal microbiome, intestinal neurons, and immune cells interact in a tripartite fashion to regulate homeostasis (Zeisel et al. 2018). Thus, microbial changes can disrupt the expression of various immune cytokines in the ENS, and inhibition of IL-6 in neurons can affect T cell numbers and phenotypes (Pratama et al. 2020). When LAT decreased IL6 expression, we hypothesized that it may have an antidepressant effect through this tripartite mechanism.

Recent evidence suggests that the deregulation of key synaptic proteins and associated dendritic and spinal complexity underlie the core pathology of depression (Heshmati et al. 2020). Brain-derived neurotrophic factor (BDNF) is a crucial neurotrophic factor involved in neuronal growth and differentiation, as well as synaptic plasticity and regeneration (Sgritta et al. 2019). Chronic defeat stress has impacts on BDNF expression in animal studies (Szuhany and Otto 2020). Another report showed alterations in the expression of BDNF and its precursor BDNF in the postmortem brain of depressed patients (Yang et al. 2016), suggesting that this protein can potentially serve as a biomarker for depression.

BDNF can promote glutamate release to act on NMDA (N-methyl-D-aspartic acid) receptors, and AMPA (α-amino-3-hydroxy-5-methyl-4-isoxazole-propionic acid) through which synaptic structures and plasticity are regulated (Afsharfar et al. 2021; Bruijniks et al. 2020). AMPA receptor is one of the main receptors mediating excitatory synaptic transmission in the brain and consists of four subunits, GluA1-4 (Ehlers 2000; Hollmann and Heinemann 1994; Man et al. 2007). Postsynaptic density (PSD) proteins embedded in dendritic spines also facilitate the transmission of glutamate signals for AMPA receptors (Harris and Weinberg 2012). Decreases in this key synaptic protein, BDNF, GluA1, and PSD95, in the PFC and hippocampus of young and adult mice, have been found in studies of CSDS, social isolation, social frustration stress, maternal deprivation, and chronic unpredicted mild stress. PSD95 and GluA1 expressions were also reduced in the depression models related to inflammation (Wang et al. 2021, 2020a, b). Additionally, BDNF levels can be altered by fecal transplants in the study of the effect of microbiota on depression (Gu et al. 2020). Thus, BDNF, GluA1, and PSD95 are useful marker proteins in studies of depression models and antidepressant effects (Castrén and Rantamäki 2010; Larsen et al. 2010; Li et al. 2009). Here, we showed that Flu and LAT can reverse the inhibitory effect of CSDS on BDNF, PSD95, and GluA1. Therefore, LAT may have an antidepressant effect by stimulating the expression of these proteins in the PFC.

The gut-brain-microbiota axis affects physiology, homeostasis, development, and metabolism (Dinan and Cryan 2017; Kelly et al. 2016). Growing research links aberrant gut microbiota to stress-related disorders including depression (Qu et al. 2017; Wang et al. 2020b), where a reduction in the abundance of α-diversity can be seen (Gloor et al. 2017; Simpson et al. 2021). In this study, we showed that the CSDS-induced alteration in gut microbiota α- and β-diversity can be reversed by LAT. Changes in the gut microbiome composition of depressed patients at the phylum level have been reported for five phyla including *Firmicutes*, *Bacteroides*, *Actinobacteria*, *Proteobacteria*, and *Clostridium*. *Bacteroidetes* and *Firmicutes* are the major bacteria communities in adults, accounting for 8.5% of fecal microbiota (Lay et al. 2005). The gut microbiome composition, including *Bacteroidetes* and *Firmicutes*, has been shown to affect cognition, anxiety, and social behavior (Robertson et al. 2017). The abundance of *Firmicutes* was decreased in depressed patients (Jiang et al. 2015; Lin et al. 2017), while *Bacteroides* was found to be higher (Aizawa et al. 2016; Chen et al. 2018). We observed similar changes in our CSDS-induced model and showed that LAT treatment can return the abundance of *Firmicutes* and *Bacteroidetes* to control levels, suggesting that the antidepressant effect of LAT may involve regulation of the gut microbiome.

Short-chain fatty acids such as propionate, acetate, and butyrate are key bacterial metabolites in the gut and are beneficial to human health (Donia and Fischbach 2015; Thorburn et al. 2014). As a G-protein-coupled receptor agonist, SCFAs can mediate intestinal epithelial signals and inhibit histone deacetylase to stabilize the nervous system. Butyrate can activate the Nrf-2 signaling pathway and promote the transcription of genes encoding antioxidant enzymes to inhibit oxidative stress (Dong et al. 2017; Li et al. 2018). These findings may explain why microbiome-modifying interventions can increase superoxide dismutase (SOD), catalase (CAT), and total-antioxidant capacity (T-AOC) levels. In addition, SCFAs regulate various intracellular and extracellular processes in the intestinal mucosa and have beneficial effects for intestinal epithelial cells and immune cells (Parada Venegas et al. 2019). Therefore, SCFAs have become popular targets in studies of depression, anxiety behavior, and cognitive function (Cox et al. 2010). The generation of SCFA has been correlated to the variation of microbial diversity and abundance (Berni Canani et al. 2016). *Firmicutes* are known to produce butyric acid in the intestines of healthy people (Soto-Martin et al. 2020). Thus, the significant elevations in butyric acid levels that we have observed with Flu and LAT treatment may be due to increased *Firmicutes* as demonstrated by the positive correlation between these two observations. Further studies will be required to determine whether LAT has the same therapeutic effect on other models of depression. Research on the role of the gut-brain axis in the development or maintenance of depression remains limited. Many reported regional or individual differences even found contrary results. Thus, more studies are required to determine the role of the gut-brain axis in depression and antidepressant drugs.

eThis study has some limitations. First of all, in this study, we only guessed through observation that it may play a role in improving depression-like behavior through regulating inflammatory factors and through the gut-brain axis, but we have not yet determined the molecular mechanism of the drug’s action. For some tests, LAT had a significant effect while Flu did not, although, with a single dose study, it cannot be concluded that LAT is more efficacious. Further investigation of the molecular mechanism and dosage will be important. Secondly, due to individual differences in the research mice, the number of mice used in the laboratory is limited, and the model is single. In order to explore the mechanism, we will continue to use other model mice to further prove the stability of the results. Third, recent studies have demonstrated the role of the subdiaphragmatic vagotomy (SDV) nerve in depression-like behavior after antibiotic treatment of mice with FMT depression-associated microbes (Wang et al. 2021, 2020a, b; Yang et al. 2017). Further study of the role of the SDV nerve in improving depression-like behavior by LAT is needed to identify drug targets.

In summary, our study showed that LAT has a comparable anti-depression effect as fluoxetine in a model of CSDS-induced depression. LAT treatment restored systemic TNF-α and IL-6 levels and the expression of prefrontal cortex proteins, BDNF, GluA1, and PSD95, that were altered by CSDS. Similarly, alterations in the abundance and diversity of the gut microbiota and the level of the short-chain fatty acid, butyric acid, can be reestablished with LAT treatment. Our experimental data suggest that the gut-brain axis may mediate the antidepressant effects of isoalantolactone.

## Supplementary information

Below is the link to the electronic supplementary material.

Supplementary file1 (PDF 50 KB)

Supplementary file2 (PDF 52 KB)

Supplementary file3 (DOCX 180 KB)

## Data Availability

Data for this study are available from the corresponding author upon reasonable request.
