# Supplementary figures and images for: Isoalantolactone relieves depression-like behaviors in mice after chronic social defeat stress via the gut-brain axis
Source: Psychopharmacology (Berl). 2023 Jul 3;240(8):1775–87. doi: 10.1007/s00213-023-06413-8 (PMC10349788; doi:10.1007/s00213-023-06413-8)

A

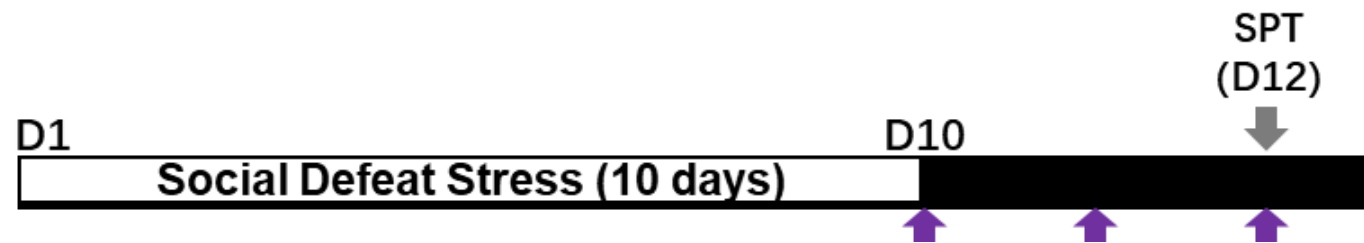

B

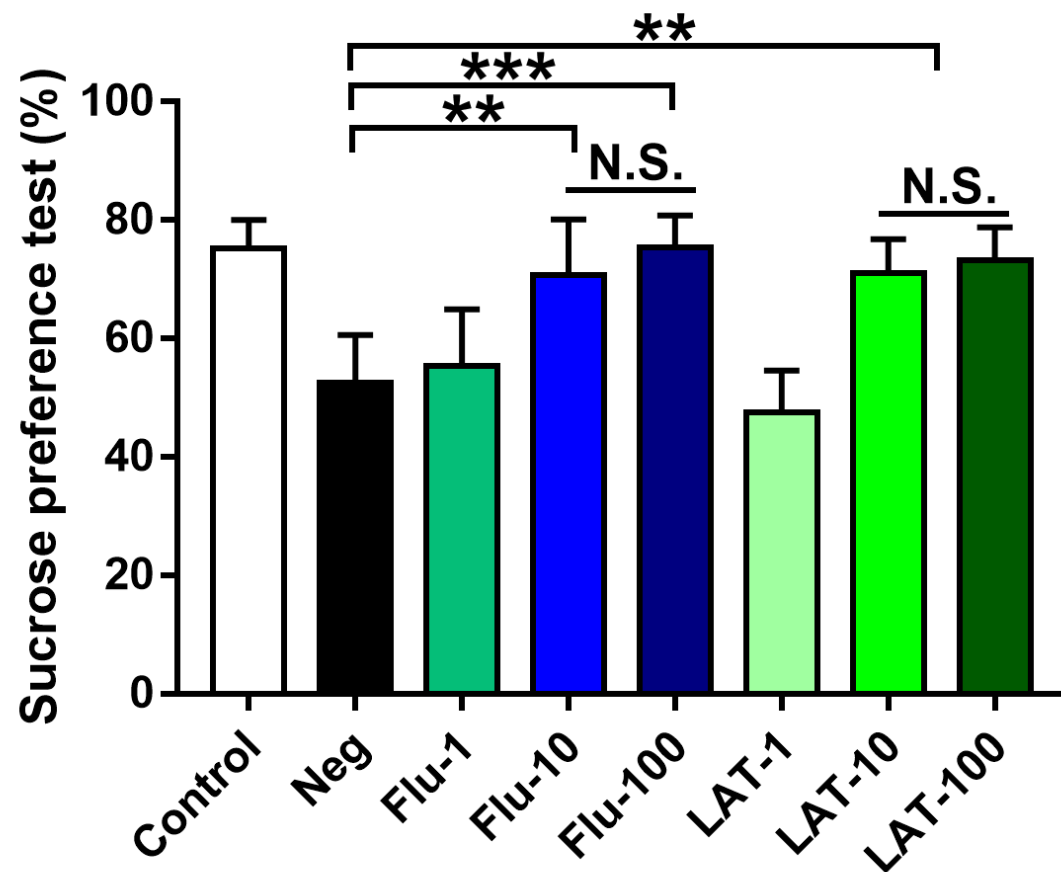

Supplement: Supplementary file 1 — Supplementary file1 (PDF 50 KB) [file 213_2023_6413_MOESM1_ESM.pdf]

C

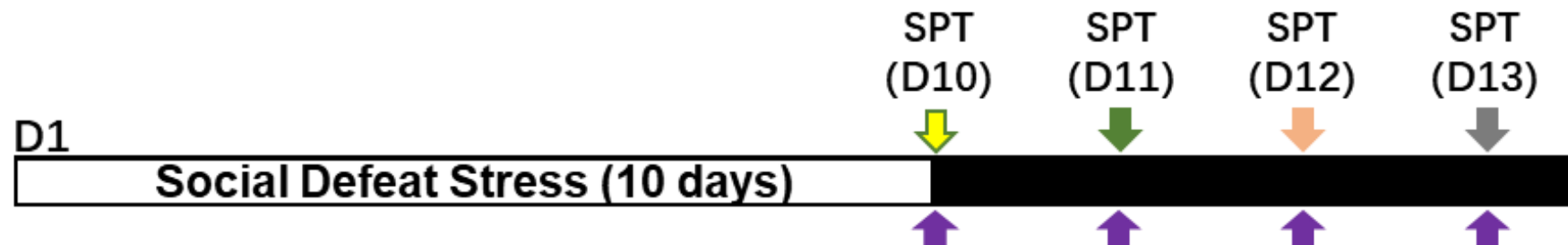

D

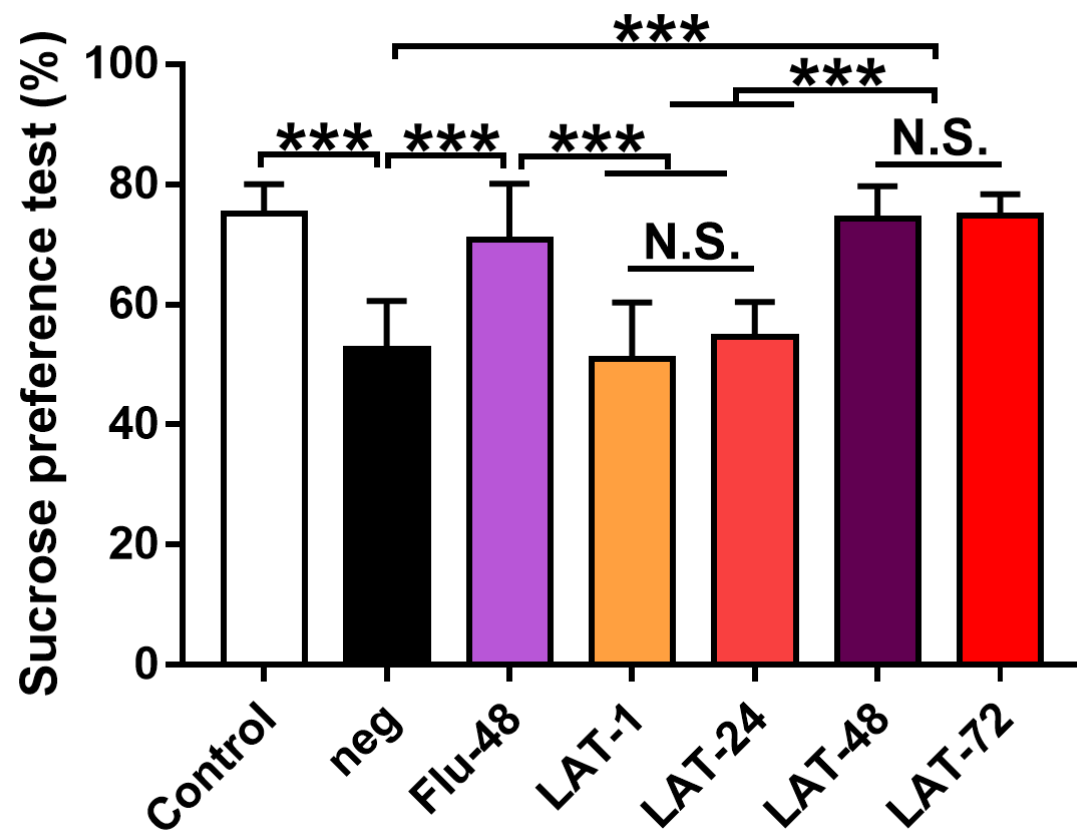

Supplement: Supplementary file 2 — Supplementary file2 (PDF 52 KB) [file 213_2023_6413_MOESM2_ESM.pdf]
