# Supplementary material for: Isoalantolactone relieves depression-like behaviors in mice after chronic social defeat stress via the gut-brain axis
Source: Psychopharmacology (Berl). 2023 Jul 3;240(8):1775–87. doi: 10.1007/s00213-023-06413-8 (PMC10349788; doi:10.1007/s00213-023-06413-8)
Supplement: Supplementary file 3 — Supplementary file3 (DOCX 180 KB) [file 213_2023_6413_MOESM3_ESM.docx]

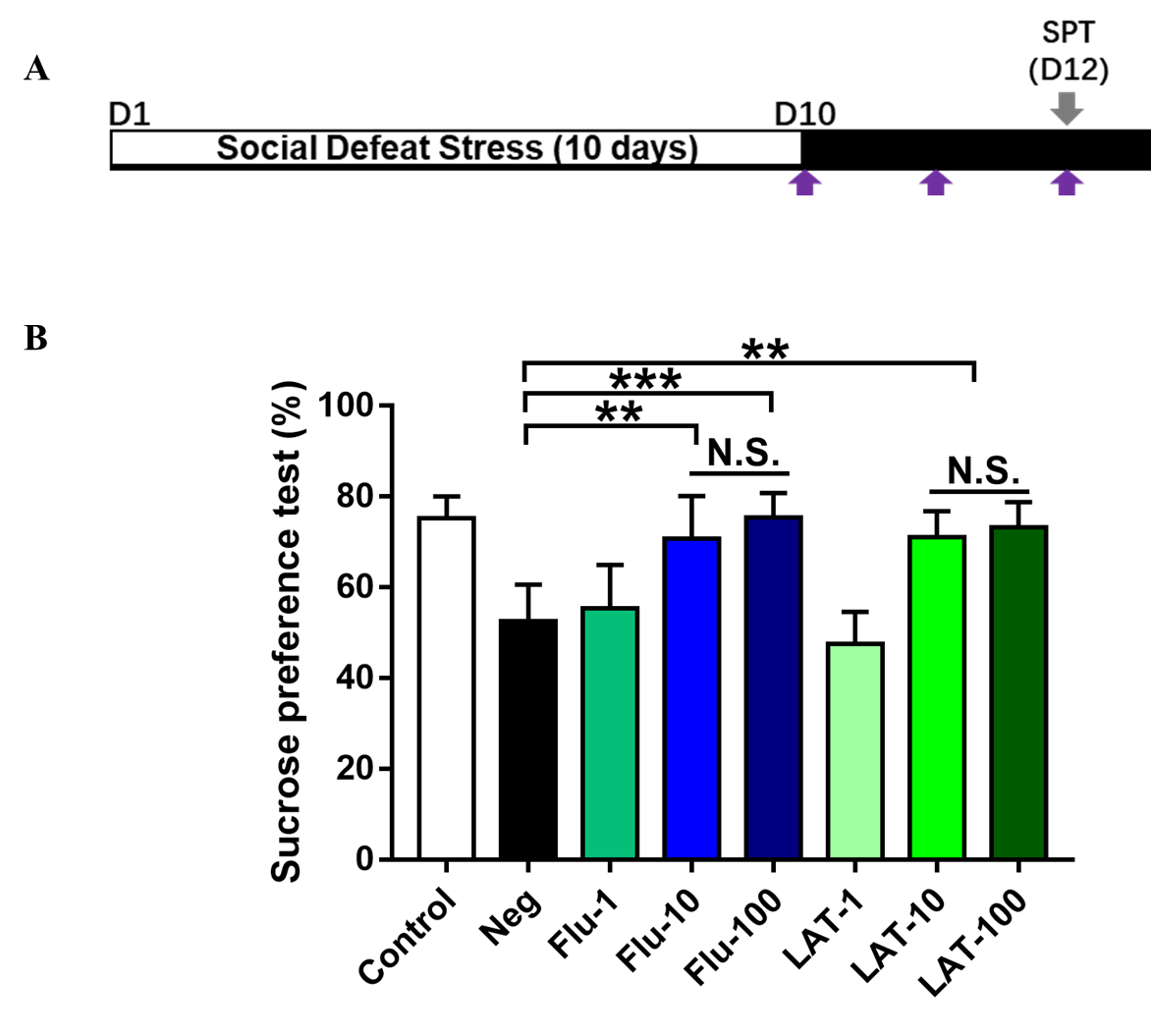


In a model of CSDS, C57BL / 6 mice were exposed to different CD1 aggressor animals for 10 minutes a day, for 10 days (Days 1-10) as previously described. On days 10, mice were administered with Flu, LAT, or CMC by oral gavage once a day at 8 am for 3 days. The eight experimental groups: (1) Control (n=5): no CSDS; (2) Neg (n =5): CSDS + 5% CMC (10 mL/kg); (3) Flu-1 (n =5): CSDS + Flu (1 mg/kg); (4) Flu-10 (n =5): CSDS + Flu (10 mg/kg) ; (5) Flu-100 (n =5): CSDS + Flu (100 mg/kg) ; (6) LAT-1 (n =5): CSDS + LAT (1mg/kg) ; (7) LAT-10 (n =5): CSDS + LAT (10mg/kg) ; (8) LAT-100 (n =5): CSDS + LAT (100mg/kg). As shown in Figure B, the results of one-way ANOVA revealed significant differences between groups on the sucrose preference (F_7,32_ =12.53, P<0.001). Post hoc comparisons indicated that compared with the neg group, Flu-10 and LAT-10 administration effectively reversed the CSDS-induced low sucrose preference (Flu: P < 0.001; LAT: P < 0.01). There was no significant difference between 10mg/kg and 100mg/kg. The results indicated that Flu and LAT 10mg/kg significantly reversed the effect of CSDS on low sucrose preference in mice 48 h after administration.





In a model of CSDS, C57BL / 6 mice were exposed to different CD1 aggressor animals for 10 minutes a day, for 10 days (Days 1-10) as previously described. On days 10, mice were administered with Flu, LAT, or CMC by oral gavage once a day at 8 am for 4 days. The seven experimental groups: (1) Control (n=5): no CSDS; (2) Neg (n =5): CSDS + 5% CMC (10 mL/kg); (3) Flu-48 (n =5): CSDS + Flu (10 mg/kg); (4) LAT-1 (n =5): CSDS + LAT (10mg/kg) ; (5) LAT-24 (n =5): CSDS + LAT (10mg/kg) ; (6) LAT-48 (n =5): CSDS + LAT (10mg/kg) ; (7) LAT-72 (n =5): CSDS + LAT (10mg/kg). Behavioral tests were performed between days 10 and 13: LAT-1 (day 10); LAT-24 (day 11); Control, neg, Flu-48 and LAT-48 (day 12); LAT-72 (day 13). As shown in Figure D, the results of one-way ANOVA revealed significant differences between groups on the sucrose preference (F_6,28_ =13.44, P<0.001). Post hoc comparisons indicated that compared with the neg group, Flu-48, LAT-48 and LAT-72 administration effectively reversed the CSDS-induced low sucrose preference (Flu-48: P < 0.001; LAT-48: P < 0.001; LAT-72: P < 0.001). There was no significant difference between 48h and 72h. The results indicated that LAT 10mg/kg significantly reversed the effect of CSDS on low sucrose preference in mice 48 h after administration.
